# Peripheral clocks and systemic *zeitgeber* interactions: from molecular mechanisms to circadian precision medicine

**DOI:** 10.3389/fendo.2025.1606242

**Published:** 2025-05-29

**Authors:** Jhommara Bautista, Sofía Ojeda-Mosquera, Dylan Ordóñez-Lozada, Andrés López-Cortés

**Affiliations:** ^1^ Cancer Research Group (CRG), Faculty of Medicine, Universidad de Las Américas, Quito, Ecuador; ^2^ Facultade de Ciencias, Campus de A Zapateira, Universidade da Coruña, A Coruña, Spain; ^3^ Instituto de Investigación Biomédica de A Coruña (INIBIC), A Coruña, Spain

**Keywords:** circadian rhythms, peripheral clocks, zeitgebers, chronotherapy, metabolic disease, cardiovascular dysfunction, neurodegeneration, cancer

## Abstract

Circadian rhythms orchestrate nearly every aspect of human physiology through a hierarchical network of clocks. While the suprachiasmatic nucleus (SCN) serves as the central pacemaker, peripheral clocks within the brain, heart, liver, gut, pancreas, adipose tissue, adrenal glands, lungs, and skeletal muscle independently regulate organ-specific functions. These autonomous oscillators, governed by transcriptional–translational feedback loops of core clock genes, align with environmental and physiological *zeitgebers* such as light, feeding, temperature, and hormones. Disruption of this temporal organization—through shift work, genetic alterations, or lifestyle factors—drives systemic misalignment, contributing to metabolic disease, cardiovascular dysfunction, neurodegeneration, cancer, and immune imbalance. This review explores the molecular mechanisms and physiological roles of peripheral clocks across organ systems, emphasizing their interplay with the SCN and *zeitgebers*. We also highlight emerging chronotherapeutic strategies that exploit circadian biology to optimize treatment outcomes. Understanding inter-organ circadian communication is key to unlocking personalized interventions and restoring systemic rhythmicity for health.

## Introduction

**Figure 1:**
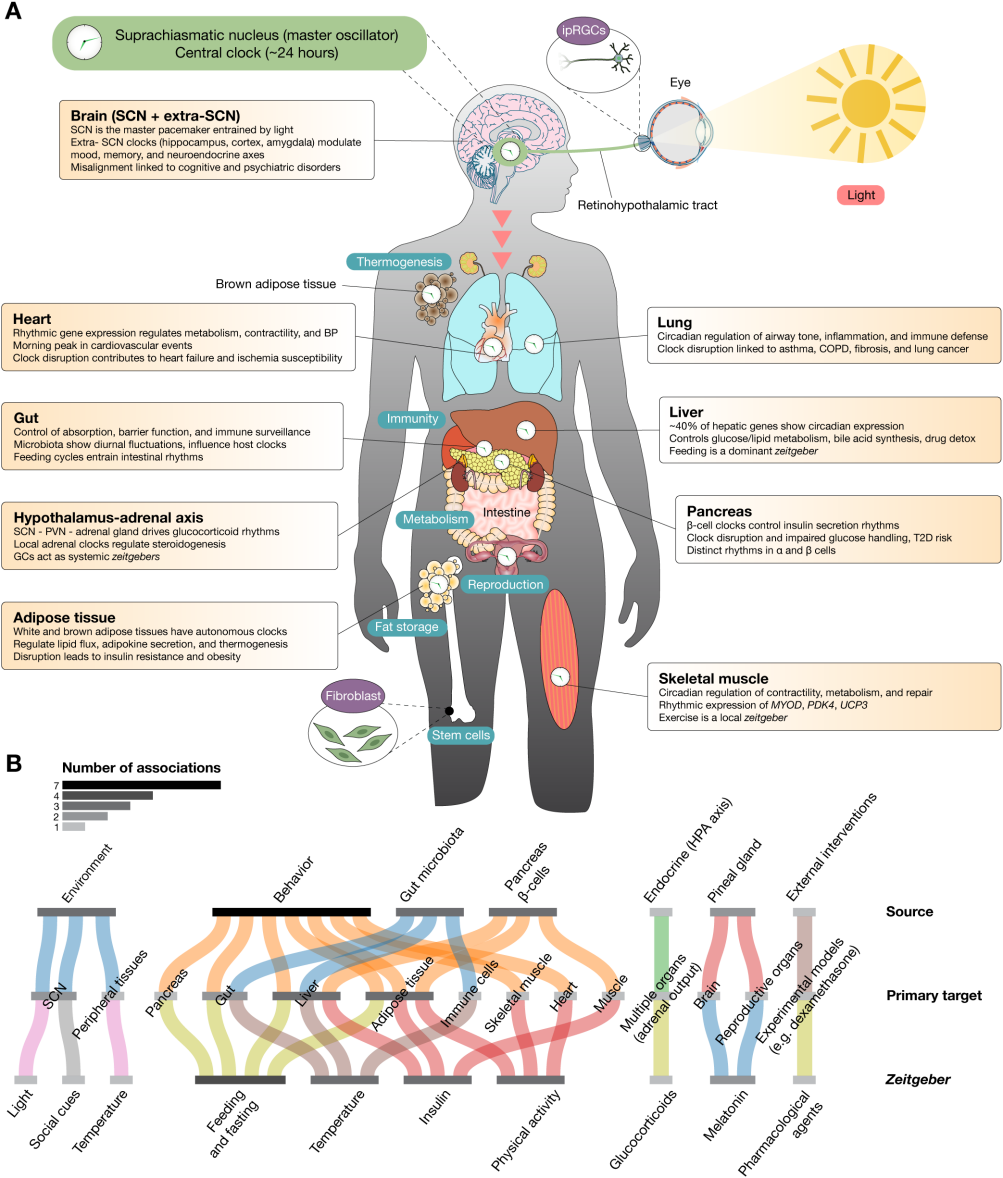
Multiorgan distribution of peripheral circadian clocks and their physiological functions. **(A)** The human body contains autonomous circadian clocks in nearly all major organs, each governed by transcriptional–translational feedback loops involving core clock genes. These peripheral clocks coordinate tissue-specific processes including metabolism, hormone secretion, immune defense, and cellular repair. Although synchronized by the central pacemaker in the suprachiasmatic nucleus via neural and hormonal signals, peripheral oscillators can also be entrained by local *zeitgebers* such as feeding, temperature, physical activity, and glucocorticoids. Key organ-specific functions are highlighted: brain clocks regulate cognition, mood, and neuroendocrine axes; cardiac clocks modulate metabolism and electrophysiology; gut clocks integrate host-microbiota dynamics; hepatic clocks control nutrient metabolism and xenobiotic detoxification; adipose clocks manage lipid flux and thermogenesis; pancreatic clocks govern insulin and glucagon rhythms; adrenal clocks drive glucocorticoid secretion; lung clocks influence airway physiology and immune defense; and skeletal muscle clocks regulate performance, energy use, and regeneration. Disruption of circadian alignment across these systems contributes to chronic diseases. **(B)** Sankey plot illustrating the associations between *zeitgebers*, primary targets, and their sources. The grayscale color scale represents the number of associations: lighter shades indicate fewer events, while darker shades represent a higher number of events. *Zeitgebers* are environmental or physiological cues that synchronize the internal biological clock (circadian rhythms) with the external environment. Primary targets refer to the main physiological systems, tissues, or molecular pathways that respond directly to zeitgebers. Sources are the origins of the *zeitgeber* signals or the systems delivering these cues. They can be external (environmental) or internal (systemic cues).

Circadian rhythms are near-24-hour endogenous cycles that govern a wide spectrum of physiological and behavioral functions, including sleep-wake cycles, hormone secretion, metabolism, and immune responses. These rhythms are controlled by a complex hierarchy of cellular clocks present in nearly every tissue, all coordinated by the central pacemaker located in the suprachiasmatic nucleus (SCN) of the hypothalamus (1, 2). The molecular architecture underlying circadian clocks is based on transcriptional-translational feedback loops (TTFLs) involving core clock genes—*BMAL1*, *BMAL2*, *CLOCK*, *CRY1*, *CRY2*, *CSNK1D*, *CSNK1E*, *NPAS2*, *NR1D1*, *NR1D2*, *PER1*, *PER2*, *PER3*, *RORA*, *RORB*, *RORC*, *and TIMELESS*—modulated by secondary regulators like *REV-ERB*s and *ROR*s (2, 3). These feedback loops drive rhythmic gene expression that synchronizes internal physiology with external or internal *zeitgebers*, including light, feeding schedules, temperature fluctuations, exercise, hormonal rhythms (e.g., glucocorticoids and insulin), microbial metabolites, and even social and pharmacological cues (1) (**Figures 1A, B**).

The SCN receives light input through intrinsically photosensitive retinal ganglion cells and synchronizes peripheral oscillators via neural, hormonal, and behavioral signals (1, 4). However, peripheral clocks possess a significant degree of autonomy and can be entrained by local cues such as feeding schedules, body temperature, and hormonal fluctuations (4–6). This decentralized and multi-oscillator structure enables organs to fine-tune their functions to specific daily demands, integrating both central and local signals to maintain systemic homeostasis (7, 8).

Disruptions in circadian synchronization are increasingly implicated in a broad array of chronic diseases, including metabolic syndrome, cardiovascular disease, neurodegenerative conditions, and cancer (1, 2, 9). Peripheral clocks in organs such as the liver, heart, pancreas, gut, adipose tissue, adrenal gland, lung, skeletal muscle, and even in non-SCN regions of the brain, independently regulate essential physiological processes. The interplay among these clocks—and between them and the SCN—provides opportunities for therapeutic interventions aimed at restoring circadian alignment. In this review, we examine the mechanisms and functions of peripheral clocks across multiple human organs and explore how their disruption contributes to disease.

## Central and extra-SCN oscillators in neurocognitive and neuroendocrine regulation

The brain houses the central circadian pacemaker in the SCN and includes autonomous oscillators in other regions such as the hippocampus, amygdala, cortex, and olfactory bulb. These extra-SCN clocks support region-specific functions, including cognition, mood regulation, sensory processing, and neuroendocrine control (8, 10). All brain clocks operate via TTFLs involving CLOCK, BMAL1, PER, and CRY proteins, generating self-sustaining ~24-hour molecular cycles (8, 10). The SCN, situated in the anterior hypothalamus, receives photic signals through the retinohypothalamic tract and orchestrates circadian rhythms via neuroendocrine, autonomic, and behavioral outputs, as demonstrated predominantly in rodent models (11, 12). Its ~20,000 neurons include distinct populations such as VIP- and AVP-expressing cells, which mediate intercellular synchronization and broadcast time cues to both brain and peripheral tissues (12, 13). In the hippocampus, circadian rhythms regulate long-term potentiation and synaptic plasticity, which are critical for memory formation. Desynchronization between hippocampal and SCN rhythms has been shown in rodents to impair learning and memory (14, 15). Similarly, local clocks in the amygdala and cortex modulate emotion and cognition, with disruptions in these rhythms, observed in both animal models and human clinical studies, being associated with psychiatric and neurodegenerative disorders (8, 12, 15). The brain’s circadian influence extends to neuroendocrine axes, especially the hypothalamic-pituitary-adrenal (HPA) axis (16, 17). Rodent studies have demonstrated that the SCN modulates glucocorticoid rhythms via the PVN and adrenal gland, while glucocorticoids, in turn, serve as *zeitgebers* for peripheral clocks (12, 18). This bidirectional relationship is further modulated by feedback from metabolic cues and hormones like melatonin and cortisol, illustrating the integrative nature of circadian timing (18). Genetic or environmental circadian disruption—via clock gene knockouts or chronic *jet lag* models in rodents—can lead to altered neurogenesis, increased neuroinflammation, and cognitive decline (8, 15). Lastly, a growing body of evidence from human clinical studies and animal models links circadian misalignment to neurological and psychiatric conditions including Alzheimer’s disease, Parkinson’s disease, Huntington’s disease, amyotrophic lateral sclerosis, epilepsy, bipolar disorder, schizophrenia, autism spectrum disorders, anxiety disorders, dementia, and depression, emphasizing the critical role of circadian synchronization in mental health (19–22).

## Temporal control of heart function, electrophysiology, and disease risk

The heart harbors an intrinsic circadian clock that regulates daily oscillations in cardiac metabolism, contractility, electrophysiology, and susceptibility to disease. While centrally influenced by the SCN pacemaker, the cardiac clock operates autonomously via TTFLs involving CLOCK, BMAL1, PER, CRY, and nuclear receptor proteins like REV-ERBs and RORs. These mechanisms have been extensively characterized in murine models and shown to regulate time-of-day–dependent gene expression in both rodent and human cardiac tissue (23–25). Cardiomyocytes and vascular cells exhibit circadian transcriptional programs that control approximately 6–10% of cardiac genes, including those involved in energy metabolism, contraction, redox homeostasis, and protein turnover (25–27). These rhythmic gene expressions prepare the heart for daily fluctuations in workload, with peak contractility, mitochondrial ATP production, and fatty acid oxidation occurring during the active phase (26, 28). In both rodent models and human observational studies, metabolic substrate use is temporally regulated: fatty acid oxidation dominates during the active phase, while glucose oxidation increases during rest (28). Disruption of these rhythms, such as through shift work or chronic circadian misalignment, impairs metabolic flexibility and contributes to pathological remodeling, including hypertrophy, fibrosis, and eventually heart failure (29). Experimental models demonstrate that repeated light-phase shifts—mimicking *jet lag*—induce diastolic dysfunction and features of heart failure with preserved ejection fraction (HFpEF). Mechanistically, this is linked to the downregulation of the CLOCK–sGC–cGMP–PKG1 axis, a signaling pathway critical for myocardial relaxation and vascular tone (29). Circadian rhythms also govern cardiovascular physiology at the systemic level. Blood pressure, heart rate, vascular tone, and cardiac output follow daily cycles, with peaks in the early active phase (23, 30). This coincides with increased sympathetic tone and renin-angiotensin activity, contributing to the observed morning surge in cardiovascular events such as myocardial infarction and stroke (23, 29, 31). Loss of synchrony between the SCN and peripheral cardiac clocks alters the timing of metabolic and electrophysiological events. Cardiomyocyte-specific deletions of *BMAL1* or *CLOCK* result in impaired contractility, mitochondrial dysfunction, and heightened sensitivity to ischemic damage (26, 30). Moreover, circadian regulation of fibrinolysis and coagulation plays a critical role in thrombotic risk. Plasminogen activator inhibitor-1 (PAI-1), which inhibits fibrinolysis, peaks in the early morning alongside elevations in blood pressure and sympathetic activity, contributing to a transient prothrombotic state (31). Chronotherapy—aligning treatment with biological rhythms—offers a compelling strategy in cardiovascular medicine. Timing the administration of antihypertensives, antiplatelet agents, or metabolic modulators may improve outcomes and minimize adverse effects (25, 32). Pharmacological restoration of circadian pathways, such as via sGC activators like riociguat, is being explored as a potential intervention for circadian-related cardiac dysfunction (29).

## Circadian regulation of gut physiology and the host–microbiota interface

The gastrointestinal (GI) tract exhibits robust circadian rhythmicity across its many functions, including nutrient absorption, barrier maintenance, immune defense, and host-microbiota interactions. These rhythms are driven by intrinsic gut clocks and modulated by external signals from the SCN, feeding behavior, and microbial activity. Most of the mechanistic evidence stems from murine models, although emerging data from human studies support similar principles (33, 34). At the molecular level, intestinal clocks operate through canonical TTFLs involving CLOCK, BMAL1, PER, and CRY proteins. These regulate circadian gene expression affecting epithelial renewal, nutrient transport, and mucosal defense (34, 35). Daily variations in stem cell proliferation, mucus secretion, and immune surveillance reflect the influence of circadian regulation and are synchronized with feeding rhythms and microbial cues (36, 37). The gut microbiota itself exhibits diurnal oscillations in both composition and function, shaped by feeding cycles and host circadian clocks (33, 35). Around 10–15% of bacterial taxa fluctuate across the day, generating time-specific production of metabolites like short-chain fatty acids (SCFAs) and bile acids. These microbial products can act as *zeitgebers*, synchronizing peripheral clocks and even influencing the phase of circadian genes like *PER2* in the liver and colon, as shown in rodent models (38, 39). Microbiota-driven rhythms are critical for gut homeostasis. Germ-free or antibiotic-treated mice show reduced clock gene expression and diminished rhythmic chromatin accessibility in gut epithelial cells (38–40). Conversely, circadian misalignment due to behavioral or environmental disruptions alters the microbial composition and metabolite cycling, leading to dysbiosis and inflammation (35, 41). Dietary patterns strongly influence gut circadian rhythms. Time-restricted feeding restores microbial and host gene oscillations even in disrupted systems (33). In contrast, high-fat or high-sugar diets dampen both microbial and host rhythms, impairing metabolic health (34, 39, 42). Protein-rich diets, however, enhance microbial rhythmicity and metabolite diversity (33). The gut clock-microbiota axis also governs immune defense. Circadian regulation of antimicrobial peptides, leukocyte trafficking, and mucosal barrier integrity coordinates with microbial dynamics to mitigate infection risk (36, 37). Susceptibility to pathogens like Salmonella varies with the circadian phase, emphasizing the importance of synchrony between host defense and microbial activity (36). Despite recent insights, questions remain about which microbial species are rhythm drivers and how non-bacterial members like fungi or archaea fit into this regulatory network (41). Translating findings from animal models to human physiology remains a key challenge.

## Metabolic coordination and chrono-pharmacology in the liver

The liver displays robust circadian rhythms tightly coupled to feeding-fasting cycles. Its peripheral clock operates through TTFLs involving CLOCK, BMAL1, PER, CRY, and nuclear protein receptors like REV-ERBs and RORs (43–45). These oscillators regulate about 40% of hepatic transcripts, including genes for glucose metabolism, lipid handling, bile acid synthesis, and xenobiotic detoxification, as shown primarily in murine transcriptomic studies (44, 46, 47). Though the SCN synchronizes liver rhythms via hormonal and neural outputs, feeding serves as a dominant *zeitgeber.* Restricted feeding can entrain liver clocks independently of light cues, demonstrating the organ’s metabolic sensitivity (45, 48, 49). The *CLOCK: BMAL1* complex activates *PER* and *CRY* expression, which in turn inhibit *CLOCK: BMAL1* in a negative feedback loop, while *REV-ERBs* and *RORs* further modulate *BMAL1* transcription (45, 47, 50). Liver-specific deletion of *BMAL1* disrupts glucose and lipid metabolism, leading to insulin resistance and hepatic steatosis (47, 48, 51). Circadian misalignment through *jet lag* or shift work, supported by both animal models and epidemiological studies in humans, exacerbates metabolic dysfunction and increases the risk of obesity and nonalcoholic fatty liver disease (NAFLD) (44, 46, 47). Chrono-pharmacology reveals time-of-day–dependent variability in drug metabolism. Hepatic expression of cytochrome P450 enzymes and inflammatory mediators like IL-6 and TNF-α fluctuates across the day, affecting responses to xenobiotics and pathogens (52). The liver also communicates with other organs: synchronized interactions with skeletal muscle and the gut optimize glucose homeostasis and energy balance. Disruption in intestinal clocks, for example, alters hepatic gluconeogenesis and lipid synthesis (49–51). Lastly, a study in clock-deficient mice shows that feeding can partially restore hepatic rhythms, though at reduced amplitude, highlighting the dual role of intrinsic clocks and behavioral cues (47).

## Temporal regulation of lipid metabolism, thermogenesis, and endocrine signaling

Adipose tissue is a circadian-regulated endocrine organ comprising white (WAT), brown (BAT), and inducible beige fat. Each type contains autonomous clocks that synchronize with the SCN and respond to local cues such as feeding, temperature, and physical activity. Most functional and molecular insights derive from rodent models (53, 54). In WAT, core clock genes regulate lipid uptake and release, adipogenesis, and adipokine secretion. Genes such as *PPARγ*, *NAMPT*, and *SREBF1* exhibit rhythmic expression, influencing lipid metabolism and insulin sensitivity. Adipokines like leptin and adiponectin are secreted diurnally in both mice and humans, modulating systemic energy balance (53, 55, 56). Disruption of WAT clocks—via high-fat diet or genetic ablation of *BMAL1* or *CLOCK*—leads to impaired adipogenesis, altered hormone secretion, and increased risk of obesity and insulin resistance (55, 56). In BAT, the circadian clock governs thermogenesis via rhythmic expression of *UCP1*, *PPARGC1A*, and fatty acid oxidation genes. These processes peak before the active phase to meet energy demands. *CLOCK* and *BMAL1* promote thermogenic gene expression, while *REV-ERBα* suppresses it. Sympathetic input from the SCN enhances BAT rhythmicity, which is diminished in circadian mutants or under chronic light exposure (57, 58). Beige adipocytes in WAT also exhibit circadian regulation. Their recruitment and thermogenic capacity decline with clock disruption, contributing to obesity in mouse models (58). Lastly, transcriptomic studies reveal that WAT gene expression follows intrinsic rhythms, with regulatory genes peaking in the morning and oxidative metabolism genes in the evening (59). Time-restricted feeding restores these rhythms and improves metabolic outcomes (60).

## Circadian control of steroidogenesis and systemic hormonal rhythms

The adrenal gland generates circadian glucocorticoid (GC) rhythms crucial for homeostasis and stress responses. While GC secretion is SCN-regulated via the HPA axis, the adrenal cortex also contains autonomous clocks essential for steroidogenesis—findings supported primarily by rodent studies (61, 62). Within the zona fasciculata, the *BMAL1:CLOCK* complex drives *PER* and *CRY* expression and regulates steroidogenic genes such as *STAR*, which mediates cholesterol transport into mitochondria. *STAR* expression follows a circadian pattern and is disrupted by adrenal-specific *BMAL1* deletion, impairing GC rhythms (63). The SCN entrains the adrenal clock through hormonal cues and sympathetic innervation via the splanchnic nerve. Light pulses can induce acute GC release independently of adrenal clocks in both rodent and primate models, reflecting direct SCN influence (64). Optimal GC rhythmicity requires both central and adrenal clocks. Disruption leads to blunted hormonal cycles and pathologies including hypertension, metabolic syndrome, and altered feeding behavior (62, 64, 65). GCs also serve as systemic *zeitgebers*, synchronizing peripheral clocks and aligning energy mobilization with activity cycles. Dysregulation impairs immune function, cognition, and cardiovascular health (66–68).

## Rhythmic regulation of lung function, inflammation, and disease susceptibility

The lung features strong circadian regulation that impacts pulmonary physiology, immune defense, and response to environmental stress. Autonomous lung clocks, composed of core TTFL components and nuclear receptors like *REV-ERBα* and *RORα*, orchestrate rhythmic gene expression in airway cells and resident immune populations, primarily characterized in rodent models (69, 70). These rhythms influence airway tone, mucus secretion, inflammation, and oxidative stress. Lung clocks also respond to systemic signals such as glucocorticoids and catecholamines, aligning pulmonary functions with behavioral cycles (69, 70). Disruption of lung clocks contributes to diseases such as asthma, chronic obstructive pulmonary disease (COPD), and fibrosis. In COPD, core clock genes like *BMAL1* and *CLOCK* are suppressed by cigarette smoke, accelerating inflammation and senescence via the MAPK pathway (71). *REV-ERBα* modulates fibrosis-related gene expression; its loss exacerbates fibrotic responses, while its activation offers therapeutic potential (72). Circadian rhythms also regulate pulmonary immune responses. Time-of-day–dependent variation in leukocyte trafficking and cytokine expression alters susceptibility to infection and inflammation (73–76). Lastly, chronic circadian disruption promotes lung tumorigenesis in KRAS-driven models through HSF1 hyperactivation (77).

## Circadian control of islet hormone secretion and glucose homeostasis

The pancreas exhibits circadian regulation in both endocrine and exocrine compartments. Core clock genes orchestrate daily rhythms in insulin secretion, glucose sensing, and islet cell function (78–80). In β-cells, autonomous clocks control insulin release in response to feeding. *BMAL1* deletion impairs glucose-stimulated insulin secretion (GSIS), reduces β-cell mass, and alters circadian gene expression (81, 82). α- and β-cells exhibit distinct rhythmic gene profiles, coordinating insulin and glucagon output across the day (81). Clock genes regulate *GLUT2*, *GCK*, and components of the exocytosis machinery such as SNAREs and calcium channels (78, 83). GSIS peaks during the active phase, optimizing nutrient handling (78). Disruption of pancreatic clocks contributes to type 2 diabetes by impairing insulin secretion and increasing resistance (80, 83). Circadian disruption also exacerbates acute pancreatitis by impairing immune timing and tissue recovery (84). Lastly, in pancreatic cancer, circadian gene expression is suppressed, and *BMAL1* deficiency in murine models enhances tumor growth and chemoresistance (85).

## Muscle metabolism, performance, and regeneration under circadian control

Skeletal muscle contains autonomous circadian clocks that govern metabolism, contractility, and repair. These TTFL-based clocks regulate the expression of genes like *MYOD*, *UCP3*, *FBXO32*, *PDK4*, and *MYH1*, affecting muscle growth, mitochondrial activity, and protein turnover (51, 86, 87). About 3–5% of the muscle transcriptome is rhythmically expressed, peaking during the active phase in mice to support performance and energy use (86–88). *BMAL1* deletion leads to muscle atrophy, impaired contractility, and altered fiber composition via disrupted MYOD and WNT signaling (88, 89). Muscle clocks can be entrained by feeding and exercise independently of the SCN. Scheduled activity resets gene expression rhythms, enhancing performance and recovery (51, 86). Coordination with liver clocks is necessary for systemic glucose tolerance. Reconstitution of *BMAL1* in both tissues restores metabolic homeostasis in otherwise arrhythmic mice (86). Lastly, circadian disruption impairs strength and regeneration and exacerbates conditions like Duchenne muscular dystrophy (DMD), where altered myogenesis and structure reflect underlying clock dysfunction (88).

## Interplay between circadian clocks across the human body

The circadian system is a hierarchically organized and dynamically coupled network of oscillators that spans the entire human body. At its apex lies the SCN, which acts as the master pacemaker, synchronizing peripheral clocks via neural, hormonal, and behavioral cues. However, each peripheral oscillator, embedded in tissues such as the liver, heart, gut, muscle, adipose tissue, adrenal gland, and lungs, retains a remarkable degree of autonomy, allowing it to respond to tissue-specific *zeitgebers* like feeding, temperature, microbial metabolites, and physical activity. These principles are supported by extensive rodent data and are increasingly validated by human studies (90, 91).

Coherence among these clocks is not maintained solely by unidirectional signaling from the SCN. Instead, circadian rhythms in peripheral tissues exhibit a reciprocal interplay that is essential for systemic homeostasis. Experimental evidence reveals that peripheral clocks exhibit organ-specific phase relationships with the SCN and with each other, and disruptions in one organ’s clock can propagate misalignment across the network (92). For instance, hepatic clocks influence pancreatic insulin secretion, and gut-derived microbial metabolites modulate hepatic gene expression via enterohepatic circulation (90, 93). Coupling mechanisms include shared hormonal rhythms, autonomic nervous system outputs, and circulating metabolites that act as systemic synchronizers. Glucocorticoids, for instance, not only reflect SCN activity through the HPA axis but also entrain clocks in peripheral tissues including the lung, liver, and adipose tissue (91, 94). Moreover, peripheral clocks themselves contribute to the feedback regulation of the HPA axis and metabolic pathways, suggesting a bidirectional flow of timing information (90, 95).

Mathematical and experimental modeling of circadian networks has further supported the notion that the circadian system behaves as a coupled oscillator network, where the stability of rhythmic output depends on robust inter-oscillator communication (91). Weak or disrupted coupling, as seen in chronic *jet lag*, shift work, or genetic ablation of clock genes, leads to phase desynchrony and dampened rhythmicity, thereby increasing vulnerability to metabolic, inflammatory, and neuropsychiatric diseases (92, 96). Understanding the interplay among circadian clocks opens new avenues for chrono-therapeutic strategies that aim to restore inter-organ synchrony. Approaches such as timed feeding, light exposure, physical activity, and pharmacological modulation of clock components are under investigation to recalibrate misaligned circadian systems in clinical contexts.

## Conclusions and future perspectives toward circadian precision medicine

Circadian rhythms are central to the temporal regulation of physiology across virtually all organs. The existence of autonomous peripheral clocks in the brain, heart, gut, liver, adipose tissue, adrenal gland, lung, pancreas, and skeletal muscle—established largely through rodent studies and increasingly supported by human transcriptomic and imaging data— highlights the widespread integration of circadian timing into cellular and systemic processes. These clocks are not only coordinated by the suprachiasmatic nucleus but are also entrainable by diverse environmental and physiological *zeitgebers*, such as light, feeding, temperature, and hormonal cues (3). The precision of this temporal network is critical for health, while its disruption contributes to a broad range of chronic diseases.

Misalignment of circadian rhythms, whether due to genetic mutations, lifestyle factors like shift work, or environmental stressors, is now recognized as a key contributor to pathologies including metabolic syndrome, cardiovascular disease, neurodegenerative disorders, cancer, and immune dysfunction (2, 7, 9). At the molecular level, these disruptions alter rhythmic gene expression, leading to impaired cellular metabolism, hormone secretion, immune responses, and stress resilience. The mechanistic insight gained from the study of peripheral clocks has also revealed tissue-specific vulnerabilities and interactions, such as the dependence of hepatic and skeletal muscle glucose regulation on synchronized oscillators (4).

Importantly, a growing body of evidence reveals that sex differences significantly modulate circadian rhythmicity, influencing both the amplitude and phase of biological rhythms across nearly all organ systems (97–99). These sex-specific variations arise from the interplay of genetic, hormonal, neuroanatomical, and epigenetic factors that affect the central pacemaker in the SCN as well as peripheral clocks throughout the body (100). Estrogens, androgens, and their respective receptors (ERα, ERβ, and AR), which are expressed in the SCN and its afferent and efferent pathways, contribute to sex-specific entrainment responses to photic and nonphotic *zeitgebers* (101). For instance, the SCN and its inputs from the retinohypothalamic tract, intergeniculate leaflet, and dorsal raphe nuclei all exhibit sexually dimorphic expression of these steroid receptors, allowing sex hormones to directly modulate the clock’s structure and output (97).

Chronotherapy—tailoring the timing of treatment to align with circadian phases—has shown promise in enhancing therapeutic efficacy and reducing toxicity. Cardiovascular drugs, chemotherapeutics, and immunomodulators can all benefit from circadian-informed administration schedules (8, 9). For example, the effectiveness of anticancer drugs has been shown to vary with the time of administration, due to circadian modulation of DNA repair, drug metabolism, and cell cycle activity (9). Furthermore, leveraging circadian biology has advanced immunotherapy strategies by identifying windows of enhanced immune responsiveness, such as time-of-day–dependent T-cell activation and cytokine production (102–104).

Moving forward, a deeper understanding of inter-organ circadian communication and *zeitgeber*-specific entrainment mechanisms will be essential for translating chronobiological principles into clinical practice. This includes identifying optimal timing for drug delivery, meal schedules, physical activity, and even surgical procedures. Technologies such as circadian biomarkers, wearable sensors, and computational modeling will support precision medicine approaches tailored to individual circadian profiles (1, 6). In conclusion, circadian biology offers a transformative framework for understanding human physiology and disease. Targeting peripheral clocks and synchronizing internal rhythms holds immense therapeutic potential across multiple domains, from metabolic and cardiovascular health to neurodegeneration, immunity, and cancer. As our understanding of these complex temporal networks deepens, chronobiology is poised to become a cornerstone of personalized and preventive medicine.
